# Three-dimensional microengineered models of human cardiac diseases

**DOI:** 10.1186/s13036-019-0155-6

**Published:** 2019-04-03

**Authors:** Jaimeson Veldhuizen, Raymond Q. Migrino, Mehdi Nikkhah

**Affiliations:** 1 School of Biological and Health Systems Engineering (SBHSE), Arizona State University, 501 E Tyler Mall Building ECG, Suite 334, Tempe, AZ 85287-9709 USA; 2 Phoenix Veterans Affairs Health Care System, Phoenix, AZ 85012 USA

**Keywords:** Cardiac, Microengineered models, Disease modeling, Stem cells

## Abstract

In vitro three-dimensional (3D) microengineered tissue models have been the recent focus of pathophysiological studies, particularly in the field of cardiovascular research. These models, as classified by 3D biomimetic tissues within micrometer-scale platforms, enable precise environmental control on the molecular- and cellular-levels to elucidate biological mechanisms of disease progression and enhance efficacy of therapeutic research. Microengineered models also incorporate directed stem cell differentiation and genome modification techniques that warrant derivation of patient-specific and genetically-edited human cardiac cells for precise recapitulation of diseased tissues. Additionally, integration of added functionalities and/or structures into these models serves to enhance the capability to further extract disease-specific phenotypic, genotypic, and electrophysiological information. This review highlights the recent progress in the development of in vitro 3D microengineered models for study of cardiac-related diseases (denoted as CDs). We will primarily provide a brief overview on currently available 2D assays and animal models for studying of CDs. We will further expand our discussion towards currently available 3D microengineered cardiac tissue models and their implementation for study of specific disease conditions.

## Introduction

Cardiac diseases (CDs) persist as the leading cause of mortality and morbidity, accounting for over 30% of deaths worldwide [1]. Notably, CDs have become the most expensive chronic disease in the United States, with $318 billion in total direct medical costs in 2015 [2]. Additionally, it is predicted that 45.1% of the U.S. population will suffer from CDs by 2035 [2]. Current research strategies employed in healthcare (e.g. pharmaceutical) industries to study CDs and to develop new therapeutic drugs mainly involve conventional two-dimensional (2D) in vitro models, such as monoculture cellular assays, as well as in vivo animal models. However, these models have significant limitations in recapitulating human pathophysiology. 2D in vitro models are limited in simulating the pathophysiology of CDs due to the high degree of complexity in structure and function of the myocardium. Specifically, these assays are unable to precisely recapitulate the complex cell-extracellular matrix (ECM), cell-cell, and tissue-level interactions. To address limitations of 2D assays, animal (e.g. mouse) models have been utilized as they are capable of complex tissue-level representation. To that end, we now have a greater understanding of the differences between mouse models and human disease, including alterations in gene expression that may affect translation of preclinical findings to human benefit [3]. However, there are many confounding factors involved in animal models, imposing challenges with transferring disease-related knowledge from these models into human pharmaceutical testing [4].

**Fig. 1:**
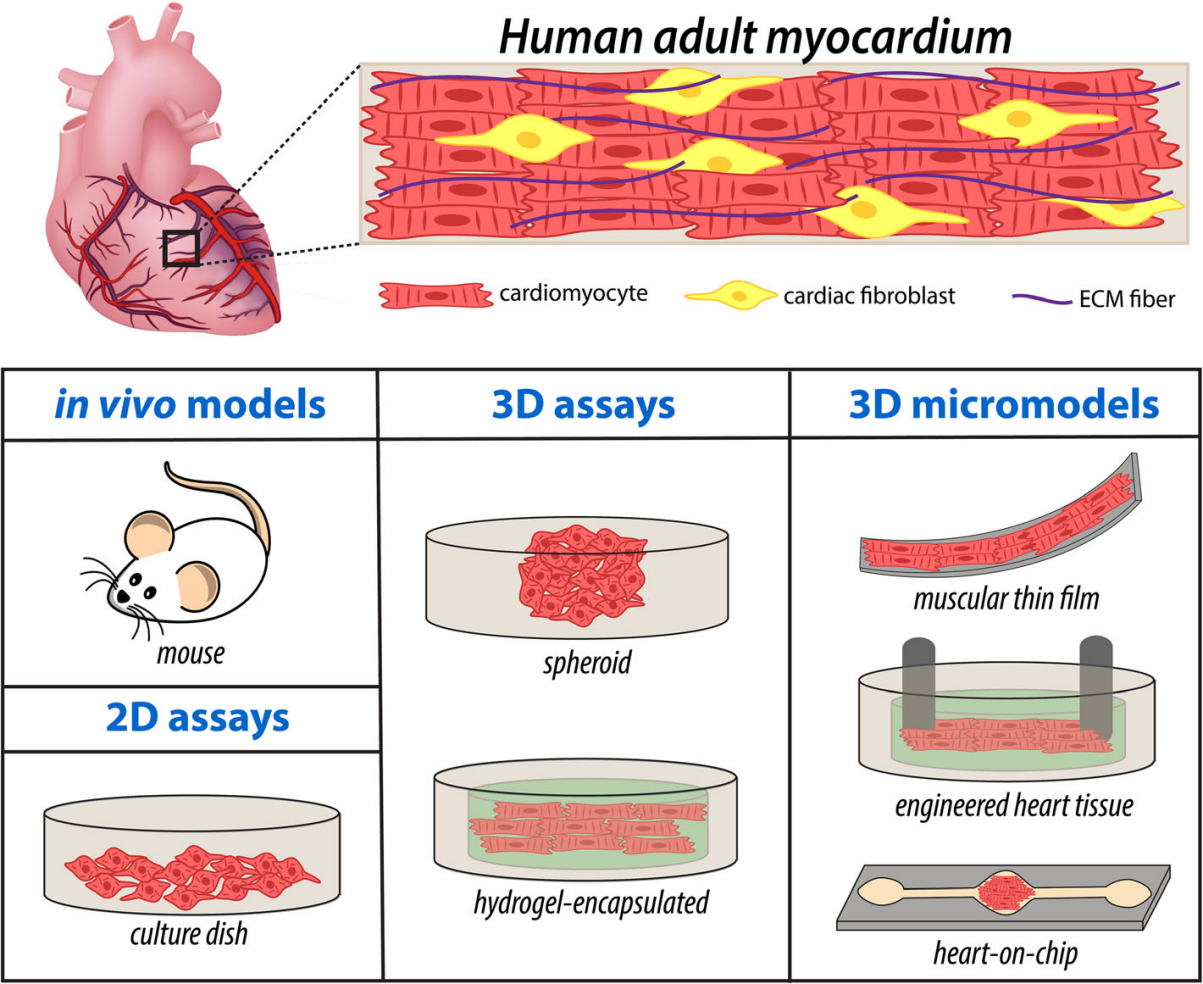
Schematic of healthy adult myocardium and examples of the different platforms implemented for cardiac tissue modeling

To improve therapeutic outcomes from CDs, attempts at addressing limitations of current 2D and animal models include creation of intricate three-dimensional (3D) cardiac tissue constructs with enhanced recapitulation of native myocardium that are useful for mechanistic studies, therapeutic discovery, and testing, with pertinent examples illustrated in Fig. 1 [5–31]. To date, a variety of techniques to create 3D cardiac tissue models have been proposed, each presenting with advantages over currently available models, including incorporation of highly controllable environments for cellular- and molecular-level studies. Recent in vitro research has also advanced the use of human-derived cardiac cells, made possible by wide availability of methods for cardiac differentiation of human stem cells to generate patient-specific and genetically-edited cardiac cells [32]. Additionally, significant emphasis on emerging biomaterials and micro−/nano- scale technologies has opened new opportunities to enhance the functionalities of engineered cardiac tissues through precise control over cell-cell and cell-ECM interactions [31, 33–36]. These models also incorporate intricacies of the native myocardium, including mimicry of anisotropic structure and accommodation of electrical and mechanical stimulation. Notably, 3D microengineered cardiac tissue models have been successfully utilized to better understand the biological basis of disease progression and enhance the efficacy of pharmaceutical testing of candidate therapeutics.

In this review article, we provide a brief overview of conventional 2D assays and animal models that have been utilized for cardiac-related disease studies. We will further review the recent progress in microengineering technologies to create 3D cardiac tissue models. A summary of current 3D diseased cardiac tissue models, with specific advantages for mechanistic biological studies and therapeutic testing, will also be provided.

## Animal models and conventional 2D assays for cardiac research

### Animal and animal-derived models

#### In vivo models

The most implemented approach for subsequent studies of CDs is through animal models [37–47]. Such models range from small to large animals, and incorporate a variety of both environmentally- and genetically-derived diseases, to study corresponding cardiac pathophysiology. Specifically, environmentally-related diseases can be studied in animal models by delivery of physical, chemical, or metabolic insults, such as through extreme change in diet or chemical administration [38, 43, 48]. Particularly, the use of animal models to study environmentally-related diseases is advantageous in the ability to recapitulate whole organ-level response to such systemic insults, an aspect that is lacking in current in vitro models. For example, to model myocardial infarction (MI), Brooks et al. chronically administered isoproterenol to mice to induce MI-related symptoms, such as impairment of diastolic function and changes in heart size, allowing for study of disease manifestation and related secondary injuries [46]. Additionally, to model acute MI, left coronary artery (LCA) ligation has been established as a technique for arterial occlusion to induce myocardial infarction in multiple types of animal models [48]. Gao et al. utilized this method to study heart remodeling and secondary pathways that occur after ligation-induced MI in mice. Significant alterations in echocardiographic characteristics, in addition to heart size and weight, were demonstrated in mice that experienced MI, thus demonstrating capabilities of animal models for systemic-level pathophysiology [49]. On the other hand, the use of transgenic animal models allows the study of genetically-derived diseases to elucidate the role of specific genes in manifestation of corresponding pathology, and potential responses to pharmaceuticals. For example, to study atherothrombotic disease, transgenic mice with mutant apolipoprotein-E have been used to recapitulate the lipoprotein profile observed in hyperlipidemia patients, causing atherosclerotic lesions to develop [47].

A particular advantage of use of animal models for study of CDs is the ability to study diseases with systemic-level pathology, and isolate the corresponding effects on cardiac function. For example, Fulop et al. incorporated Zucker diabetic fatty (ZDF) rats to determine if development of Type 2 diabetes negatively affects cardiomyocyte (CM) function [50]. Their findings unveiled that contraction, relaxation, and calcium handling characteristics were impaired for CMs isolated from 22 week-old hyperglycemic ZDF rats as compared to both 6-week old hyperinsulinemic ZDF rats and healthy age-matched controls. Thus, the use of the ZDF rat models delineated specific cardiac-related effects of diabetes.

In vivo animal models have provided fundamental knowledge into the biology of cardiac disease, and correspondingly have served as helpful models for the translation of observed pathology into potential pharmaceuticals [37–47]. However, the inability for precise control at molecular- and cellular-levels hampers the amount of mechanistic information that can be gained from these models. Incorporation of cardiac tissues within in vitro models has enabled the direct mechanistic studies of CDs that complement the knowledge gained from in vivo models for enhanced clinical translation of disease-related findings.

#### Neonatal rat cardiomyocytes in in vitro models

There have been a vast number of in vitro models to date utilizing neonatal CMs derived from animals for cardiac tissue engineering due to the ease of access and availability of these cells [31, 35, 36, 51–57]. For example, Zimmermann et al. demonstrated the utility of mechanical stretch in an in vitro model to generate aligned cardiac tissue from neonatal rat CMs and collagen hydrogel that resembles the structure of the native myocardium [52]. Saini et al. developed cardiac micro-tissues, with variable geometrical features and CM to cardiac fibroblast (CF) ratios within gelatin methacrylate (GelMA) hydrogel, to assess the role of tissue confinement and co-culture ratio on functionalities of the engineered tissues [31]. Their findings demonstrated that supplementation of CMs with CFs enhanced the tissue structure and protein expression, in addition to delineating the prominent role of architecture on tissue formation. These in vitro models have also been advanced for elaborate cardiac tissue studies, with an emphasis on modeling CDs [58–69]. For example, Mosadegh et al. created a 3D model for cardiac ischemia using rat neonatal CMs within a paper-based platform [68]. Incorporation of cell culture within this in vitro model allowed for establishment of an oxygen gradient and the subsequent study of its effect on encapsulated tissue. Particularly, CF migration was observed against the oxygen gradient, in response to signaling from CMs, providing pertinent information about the intercellular mechanisms that occur during ischemia. Despite the significance of tissue model systems developed using animal-derived cardiac cells, the discrepancies inherent between animal and human physiology significantly limits the translation of knowledge gained from these studies to implementation into the clinic [4]. To complement these models, in vitro models that provide a tunable microenvironment for precise biological studies are utilized with incorporation of human-derived cells for further insight into CD research for translational applications.

### Human PSC-CMs (pluripotent stem cell-cardiomyocytes) in 2D monolayer assays

Due to difficulties inherent in the isolation of human adult cardiomyocytes (CMs), their use within in vitro assays to date has been limited. The advent of CM differentiation from human pluripotent stem cells (hPSCs), including induced pluripotent (hiPSCs) and embryonic (hESCs), has introduced a potentially unlimited source of human cardiac cells for use within in vitro assays for disease modeling [70–75]. Cardiac diseases arise in one of the following ways: through genetic predisposition, acquired or both. Therefore, methods for disease modeling generally fall into one of the following categories to highlight the different methods of disease induction: 1) either diseased cardiac cells are directly incorporated into these in vitro models, or 2) healthy CMs are subjected to external insults to model the role of environmental impact in disease etiology. This review will focus on models derived via the former approach, through stem cell-based techniques that allow for derivation of patient-specific and/or genetically-edited cardiac cells for precise modeling of disease manifestation. Specifically, to generate such diseased cardiac cells, hPSCs are generally either reprogrammed from patient-derived fibroblasts, or genetically modified from wild-type (WT) hPSCs, to produce the cells with diseased genotype for CM differentiation and subsequent incorporation into cardiac models [76]. In the following sections, the use of these cells within 2D assays will be discussed.

#### Patient-derived hiPSC-CMs

Patient-derived hiPSC-CMs have provided enormous potential for a wide variety of disease modeling applications [74]. Long-QT syndrome (LQTS), a disease characterized by prolonged ventricular repolarization phase, often leads to sudden cardiac death in afflicted patients [77, 78]. Moretti et al. identified a missense mutation (R190Q) in KCNQ1, a gene that encodes for ion channels that generate the slow outward potassium current I_Ks_, among patients with LQTS type 1 (LQTS1). In order to elucidate mutation-related mechanisms among afflicted cardiac cells, Moretti obtained skin fibroblasts from LQTS1 patients with this mutation, performed hiPSC reprogramming, and then differentiated these pluripotent cells into CMs. Electrophysiological analysis of these CMs highlighted altered activation and deactivation properties of potassium ion channels. Subsequent stimulation through isoproterenol demonstrated an increase in action potential duration, worsening the pathophenotype in LQTS1. This finding demonstrated a possible relationship between abnormal potassium current channels and onset of sudden cardiac death, corroborating the clinical finding that fatal arrhythmias are preceded by increased sympathetic tone in patients with LQTS1 [79, 80]. Pretreatment of these cells with propranolol (a nonselective beta-blocker) blunted the effects of isoproterenol, thereby serving to protect these diseased cells from catecholamine-induced tachyarrhythmia [81]. Overall, this study highlighted the utility of patient-derived hiPSC-CMs in mechanistic-level studies and potential therapeutic testing.

Pompe disease, a metabolic disorder defined by a mutation in the acid alpha-glucosidase (GAA) gene, results in heart failure in a majority of affected patients by 18 months of age [82]. To study the relationship of this mutation with cardiac function, Huang et al. obtained skin fibroblasts from patients with mutations in GAA, performed hiPSC reprogramming and subsequent CM differentiation, and incorporated these CMs within in vitro 2D models [82]. The patient-derived hiPSC-CMs recapitulated classic Pompe disease phenotypes, such as high levels of glycogen and ultrastructural defects, and responded to administration of recombinant GAA, a treatment commonly prescribed for Pompe disease. However, the CMs failed to exhibit dramatic autophagic abnormalities, a major component of disease pathology in Pompe disease, which could potentially be attributed to absence of a 3D complex structure and microenvironment that exist in native myocardium.

Timothy syndrome (TS) is a specific form of LQTS long QT syndrome, a disorder defined by prolonged QT intervals arising from a missense mutation in the L-type calcium channel, Ca_v_1.2, that leads to arrhythmia [83]. Yazawa et al. derived hiPSC-CMs from TS patients, in junction with in vitro 2D culture, in order to study the molecular- and cellular-level properties of TS [84]. The TS hiPSC-CMs demonstrated abnormal electrophysiological properties, including irregular calcium handling and prolonged action potential duration. To test potential therapies, the researchers supplied Roscovitine, a cycline-dependent kinase inhibitor shown to increase voltage-dependent inactivation of Ca_v_1.2 channel, and demonstrated that many of these abnormal characteristics were rescued. Despite these advancements, using patient-derived cells to study a disease makes it difficult to capture all of the various facets of the available disease phenotypes, with great interpatient heterogeneities that render study of mechanisms directly related to a genetic mutation difficult to accomplish.

#### Genetically-induced diseased hPSC-CMs

To bypass inter-patient heterogeneity and specifically identify the role of certain mutations/genes in disease pathology, gene-editing, generally in the form of CRISPR (clustered regularly interspaced short palindromic repeats)/Cas9 technology, has been applied to hPSCs, which are then differentiated into CMs and incorporated into cardiac models for mechanistic investigations [85].

Type-2 Ryanodine receptors (RyR2) release calcium through a calcium-induced mechanism in the sarcoplasmic reticulum, and its mutation has been found in a majority of cases of catecholaminergic polymorphic ventricular tachycardia type 1 (CPVT1), with more than 150 associated mutations demonstrated [27]. For instance, Wei et al. used CRISPR/Cas9 to introduce point mutations in WT RyR2 of hiPSCs, then differentiated these cells into CMs [86]. Calcium handling and spontaneous beating properties were compared of the gene-edited cells to patient-derived hiPSC-CMs with the disease-associated mutation, F2483I. This approach enabled precise study of specific mutations among cells that are isogenic to specifically highlight the role of RyR2 in disease pathology, corroborating similar findings in patient-derived hiPSCs with mutated RyR2.

Additionally, gene-editing techniques have been integrated with patient-derived hiPSCs to validate the pathogenicity of a genetic variant, through correction of the hiPSCs through genome editing. For example, Liang et al. derived hiPSC-CMs from patients with Brugada syndrome (BS), a disorder associated with ST-segment elevation that leads to ventricular fibrillation and sudden cardiac death, and analyzed the cardiac pathologies, such as abnormal calcium transients [87]. To study the implication of the SCN5A variant, CRISPR/Cas9 was used to introduce correct SCN5A into the cells derived from BS patients. Correction of this genetic variant resolved many of the irregularities in the electrical profile of the CMs, such as improved peak-to-peak interval variability, highlighting the importance of this gene in pathological onset.

In summary, animal models and 2D in vitro assays constitute appropriate platforms for CVDs research. However, human native myocardium has additional structural and functional complexities, in regard to anisotropic architecture, ECM and cell-cell interactions, that are not replicated by 2D in vitro models. Incorporation of higher complexity models that better mimic human myocardium could potentially improve recapitulation of disease pathophysiology. In the next section, we will highlight some of the recent advances in development of 3D biomimetic cardiac tissue models through the use of microengineered technologies and advanced biomaterials.

## Microengineering of 3D cardiac tissue models

**Fig. 2:**
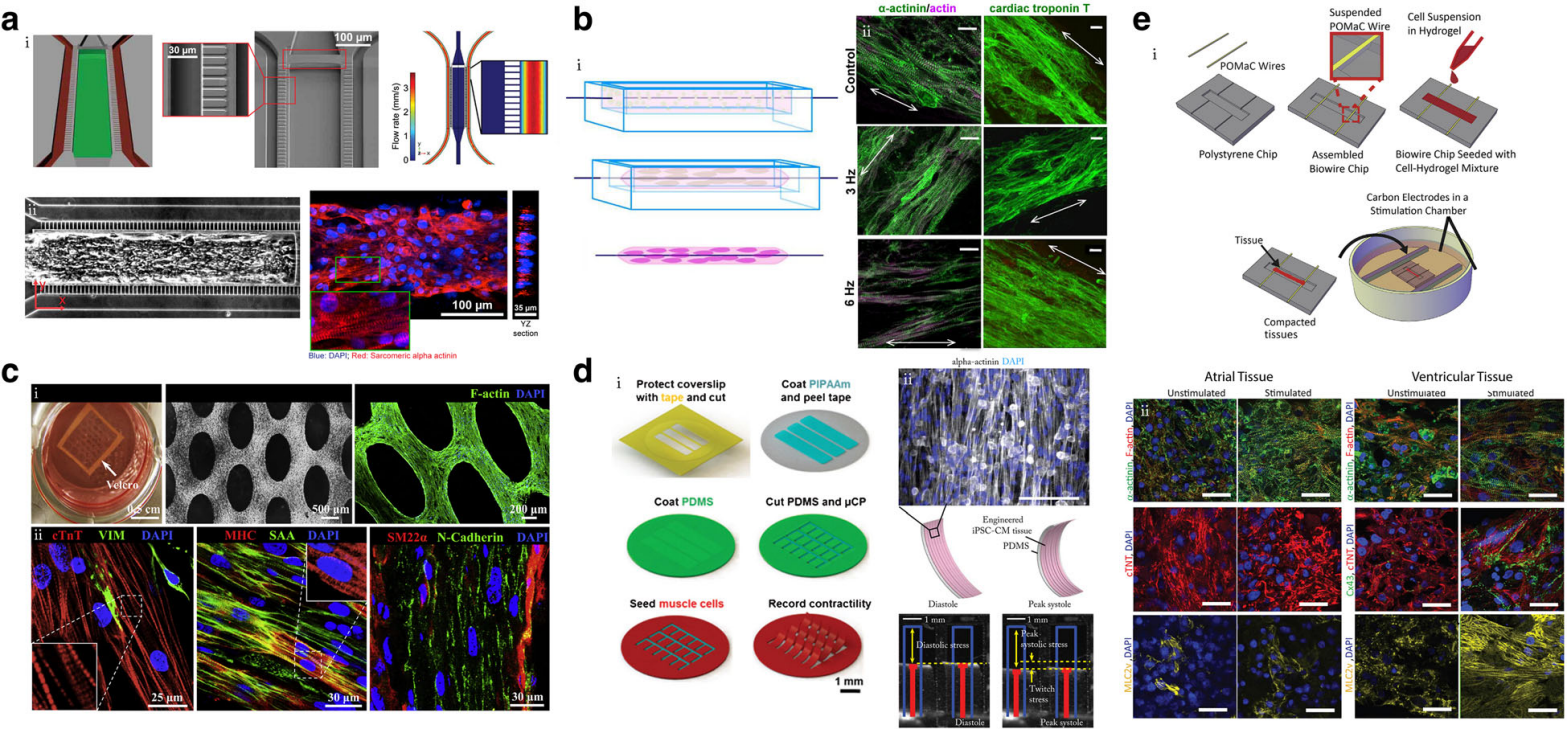
3D microengineered models of healthy cardiac tissue. **a**) i: Microfluidic channel with endothelial-like borders for cardiac tissue culture. ii: Phase contrast and cardiac-specific marker immunofluorescent staining of cultured tissue within microfluidic platform, reprinted with permission from [22]. **b**) i: Schematic of cardiac tissue culture around surgical suture. ii: Cardiac-specific marker immunofluorescent staining of tissues with and without electrical stimulation, reprinted with permission from [12]. **c**) i: Phase contrast and cytoskeletal staining of cardiac tissue formed within engineered patches. ii: Cardiac-specific and other cell-specific marker immunofluorescent staining of 2-week old cardiac tissue patches, reprinted with permission from [29]. **d**) i: Schematic of the process of engineering MTFs, reprinted with permission from [26]. ii: Cardiac-specific marker immunofluorescent staining of tissues cultured on MTFs and representation of tissue contractility measurements, reprinted with permission from [27]. **e**) i: Schematic of process for engineering Biowire II. ii: Cardiac-specific marker immunofluorescent staining of atrial and ventricular tissues, either under electrical stimulation or not, reprinted with permission from [98]

There is now increased use of 3D cardiac models that provide greater freedom in complex tissue-level interactions in addition to the incorporation of critical physiologic conditions such as whole-tissue electrical stimulation and establishment of precise gradients. Specifically, the integration of methods such as photolithography, soft lithography, and 3D printing along with advanced biomaterials and human CMs has enabled the emergence of various biomimetic 3D human cardiac tissue microengineered models [5–31, 62]. The complexity of these 3D microengineered models continues to advance for further physiological relevance, including methods to induce cardiac tissue maturation through use of co-culture of multiple cell types, incorporation of surface topography, and electrical and/or mechanical stimulation [88]. Engineered cardiac tissue models within microfluidic platforms have the advantage of providing highly controllable fluid flow to model the vasculature within the native heart, in addition to increased throughput due to significant reduction in necessary reagents and cells through platform miniaturization [89]. Mathur et al. incorporated hiPSC-CMs into an ECM-coated straight microfluidic channel, composed of polydimethylsiloxane (PDMS), with bordering arrays of microposts to serve as endothelial-like barriers for nutrient and drug diffusion [22] (Fig. 2a). To validate the model, therapeutic agents administered via the media channels served to model intravenous drug administration and expected responses were observed from the aligned hiPSC-CM tissue layer, better modeling the tissue-scale response than other cellular-level studies. Recapitulation of cardiac response in addition to the ability to control external inputs such as drug administration, while measuring relevant output such as contractile response demonstrate the utility of this platform in 3D cardiac tissue modeling.

The electrophysiological, phenotypic, and genotypic signatures of CMs generated from differentiation of hPSCs demonstrate the immature state of these cells [90], therefore multiple strategies have been implemented to enhance their maturation to better model adult human cardiac cells. For example, electrical stimulation has been incorporated into 3D microengineered models to allow electrophysiological-related studies, as well as to promote maturation of cardiac tissue [12, 91]. Nunes et al. cultured 3D hydrogel-encapsulated cardiac tissues along a surgical suture, and supplied electrical field stimulation, through submersion of tissue constructs within an external stimulation chamber, to enhance maturation of the cardiac tissues, denoted as Biowires (Fig. 2b). Stimulated cardiac tissues displayed greater maturation than their non-stimulated counterparts, as shown by increased myofibril ultrastructural organization and changes in both electrophysiological and calcium handling characteristics. Additionally, the Biowire models responded to physiological activation through β-adrenergic stimulation, as evidenced by increased frequency of spontaneous beating, thereby recapitulating clinical observations of the native myocardium. These findings demonstrate the usefulness of electrical stimulation to mature hPSC-CM-derived tissue, enhancing the physiological relevance of the model.

Another method to enhance maturation of hPSC-CM tissue is through cellular alignment to mimic the highly anisotropic nature of native myocardium. Zhang et al., through standard soft lithography, fabricated a tissue-engineered patch with surface topography, in the form of staggered hexagonal microposts of precise spacing and geometry, to induce alignment of hPSC-derived cardiac tissue [29] (Fig. 2c). The aligned, anisotropic structure of the encapsulated cardiac tissue, when compared to monolayers of isotropic tissue of identical cellular composition, demonstrated increased maturity as evidenced by a higher ratio of expressed MLC2v/MLC2a and longer striated sarcomeres. Various other works from this group have also demonstrated the merit of topographical features in creation of physiologically relevant human cardiac tissue constructs with enhanced maturation that better represent the native myocardium [29, 92, 93].

To better study the functionality of engineered cardiac tissue, methods to allow for measurement of the contractility of the tissue have been explored [19, 40, 47, 52]. One technique, muscular thin films (MTFs), involves the use of a flexible PDMS thin layer that deflects into a 3D formation during contraction of cultured CMs [27, 76], with distance of deflection reflecting force of contraction (Fig. 2d). The layer is microcontact patterned with fibronectin to induce an anisotropic formation of the CM tissue that synchronously contracts uniaxially, causing the deflection of the MTF in one direction, thus enabling the calculation of contractile force generation from the entire tissue. MTF technology has been incorporated with many cell types for tissue formation [9, 15, 94, 95], including cardiac, to elucidate alterations in tissue contraction from tissue-specific insults that influence contractile properties.

In another method to measure mechanical force of 3D cardiac tissue, the incorporation of elastic deformable silicone microposts allowed direct measurement of force during tissue contraction [20, 96]. Mannhardt et al. produced highly anisotropic hESC-derived cardiac tissue around elastomeric silicone microposts with organized sarcomeres, denoted as engineered heart tissue (EHT). Through gene expression analysis, they demonstrated physiological relevance of the model in the upregulation of cardiac markers, including MYH7, for conditions cultured within the platform compared to cardiac-differentiated embryoid bodies. Additionally, they validated the ability of the EHTs to study the effect of various inotropic modulators, i.e. calcium, isoprenaline, and ryanodine, on tissue contractility through average contraction peaks and contraction kinetics. Calculation of these metrics after drug administration, clinically-relevant, expected changes in contractility were demonstrated. The ability of these models to study the contractility of engineered cardiac tissues presents a significant advantage in assessing function in addition to structural and biochemical changes. Correspondingly, various methods that are incorporated to mature stem cell-derived cardiac tissues serve as a great advantage of these models over standard 2D in vitro assays for studies on CDs.

A majority of available stem cell differentiation protocols result in generation of ventricular-specific CMs, that are usually incorporated in the aforementioned models. As the different chambers in the heart have largely different electrophysiological signatures [97], there have been strides to incorporate both ventricular and atrial CMs within these models, through chamber-specific directed differentiation protocols. For example, Zhao et al. demonstrated a chamber-specific cardiac tissue platform, denoted as Biowire II, with sustained electrical conditioning both to mature encapsulated cells and provide distinctive pacing regimes for the different types of CMs (Fig. 2e) [98]. Specifically, the Biowire II model incorporated flexible wires within an array of microwells for cardiac tissue attachment, cellular compaction, and alignment. Atrial and ventricular tissues were formed separately, and corresponding electrical conditioning was applied for an extended period (up to 42 days). The stimulated cardiac tissues were then analyzed with comparison to their non-stimulated counterparts. In the conditions with applied electrical conditioning, they found that the different chamber-specific tissues mapped to their corresponding cardiac region gene expression patterns. This platform demonstrated its physiologic relevancy to the human native myocardium through incorporation of both atrial and ventricular CMs, in addition to enhanced maturation of CMs through sustained electrical conditioning.

## The application of 3D microengineered cardiac tissues for disease modeling

In the past few years, significant progress in establishing biomimetic, clinically relevant healthy 3D cardiac tissue models has been accomplished. In the next section, we will outline some of these currently available 3D cardiac microengineered platforms that have been successfully used for cardiac disease modeling.

To model heart failure caused by neurohumoral overstimulation, Tiburcy et al. utilized EHT technology for hPSC-CM tissue formation, then administered either norepinephrine and/or endothelin-1 over 7 days [30]. Long-term administration of norepinephrine (NE), an adrenoceptor agonist, induced CM hypertrophy as demonstrated through contractile dysfunction of the EHT, which proved unreceptive to rescue with isoprenaline in conditions with chronic application of 1 μM NE. This finding demonstrates β-adrenergic desensitization of the hypertrophied tissues, thereby corroborating the clinical phenotype often observed in patients with heart failure. This study demonstrated the successful induction of a pathophenotype through chronic application of an external insult to a healthy 3D cardiac tissue.

**Fig. 3:**
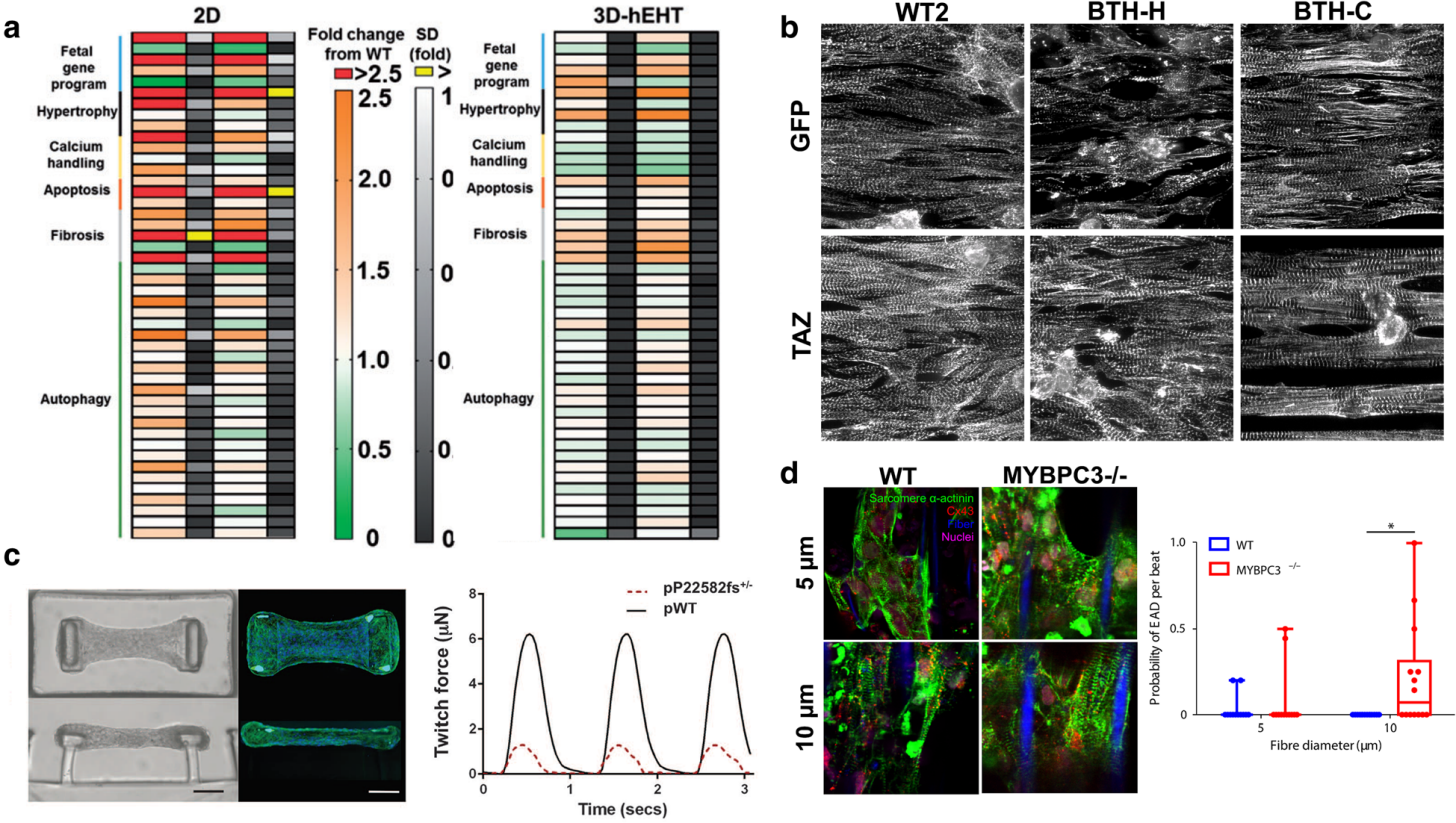
3D microengineered models of cardiac diseases. **a** Relevant gene expression changes in 2D and 3D-EHT cardiac tissue models of hypertrophic cardiomyopathy, reprinted with permission from [102]. **b** Sarcomere organization of cardiac tissues from patient-derived and gene-edited cells for modeling of BTHS with response to TAZ restoration, reprinted with permission from [27]. **c** Representative image of CMTs and twitch forces of CMTs from WT and patient-derived cells to study dilated cardiomyopathy, reprinted with permission from [103]. **d** Cardiac-related marker expression and electrical activity of WT and gene-edited cardiac tissues within constructs of different sized fibers, reprinted with permission from [100]

Mutations in myosin-binding protein C cardiac isoform (MYBPC3), the accessory protein of the sarcomere A-band, have been found in various types of cardiomyopathy [99], however the relationship between these mutations and disease onset remains unknown. To investigate the hypothesis that physiologic stress exacerbates disease symptoms in patients with these mutations, Ma et al. generated cardiac tissues from CRISPR/Cas9-edited hiPSCs deficient in MYBPC3 to identify the gene-specific response to mechanical stress [100]. Specifically, two-photon polymerization was used to fabricate filamentous matrices of different sized parallel fibers, with thicker fibers exhibiting higher mechanical resistance. Cardiac tissues composed of WT hiPSC-CMs exhibited adaptation to mechanical load alterations through changes in contraction velocity and force, mimicking the behavior of the native myocardium. Although the structural properties of tissues composed of mutated MYBPC3-dervied CMs appeared similar to the WT cardiac tissues (Fig. 3d), when exposed to higher mechanical resistance, the mutated CMs exhibited increased probability of early after-depolarizations (EADs) than the WT counterparts. These results were in fact consistent with the clinical finding that patients with cardiomyopathy are more prone to EADs. Therefore, the incorporation of a substrate with adjustable stiffness enabled assessment of tissue-level response to mechanical stress, highlighting the possible connection between onset of cardiomyopathy and mutations in MYBPC3.

3D tissue models have been also integrated with genetically-edited human-derived cardiac cells to create complex 3D diseased tissue models. For example, hypertrophic cardiomyopathy (HCM) has been widely studied because of its heterogeneity, as evidenced by the fact that half of the patients with HCM have mutations in one or more of > 20 sarcomeric genes [101]. In order to better understand the role of various known mutations in HCM, Mosquiera et al. produced 11 different variants of the HCM-causing mutation cC9123T-MYH7 in 3 different hPSC lines via CRISPR/Cas9 gene-editing [102] (Fig. 3a). After CM differentiation, the cells were incorporated into EHT models and functionalities of the different variant-derived hPSC-lines were compared to their 2D counterparts. Through transcriptomics of the tissues, opposing trends were demonstrated between 2D and 3D culture formats. Specifically, there were decreases in expression of genes involved in calcium handling and less pronounced changes in apoptosis and autophagy in the 3D tissues as compared to 2D assays. Incorporation of these gene-edited hiPSC-CMs within EHTs also allowed measurement of tissue contraction, revealing reduced contraction force and increased contraction time in tissue formed from mutated hiPSC-CMs. This study demonstrated the merit of a 3D platform over conventional 2D for disease modeling applications. Additionally, the use of various gene-edited hiPSC-CMs enabled the study of cardiac function pathology that is directly attributable to specific mutations, an advantage over the genetic heterogeneity present in patient-derived CMs. However, many works tend to use both gene-edited and patient-derived hiPSC-CMs to understand both disease-specific and patient-specific mechanisms of disease progression at the tissue-level [27, 103].

Wang et al. for instance investigated both patient-derived and genetically-engineered hiPSC-CMs of Barth syndrome (BTHS), a syndrome characterized by mitochondrial myopathy from an X-linked mutation in tafazzin (TAZ). After assessment of structural irregularities in both types of diseased CMs, cells were incorporated into MTF technology to create a 3D cardiac tissue disease model to highlight the specific contractile mechanisms that are affected in CMs with these mutations [27]. Through electrical stimulation of the MTFs, they successfully calculated radii of curvature, diastolic and peak systolic stresses, and twitch stress to quantify differences in tissue contractility (Fig. 3b). Both the BTHS-derived and gene-edited heart tissues demonstrated significantly reduced twitch and peak systolic stresses than controls, recapitulating the BTHS myopathic phenotype in an in vitro model. They further studied whether the functionalities of the disease tissues could be restored through treatment with TAZ synthetic chemically modified mRNA (modRNA). Upon TAZ modRNA administration, they observed restoration of contractile function, demonstrating the utility of this approach in reversing the myopathic disease phenotype. Particularly, incorporation of these cardiac cells within MTFs allowed the study of contractility to highlight cardiac dysfunction in BTHS in a highly controllable 3D format that is not possible in animal models or 2D in vitro assays. In a similar experimental format, a study by Hinson et al., investigated the role of mutations of the sarcomeric protein, titin, in dilated cardiomyopathy using hiPSC-CMs from patients with titin-truncating variants (TTNtvs) [103]. Incorporation of elastomeric microposts, known as microarray post detectors (mPADs), led to formation of an aligned cardiac microtissue (CMT) [6] (Fig. 3c). The microposts contained embedded fluorescent microbeads that allowed for computerized cantilever deflection tracking, providing a quantitative, objective method to measure the contraction force generated by the CMTs. They further used CRISPR/Cas9 technology to induce similar mutations of the titin gene within CMTs. In both types (i.e. patient vs genetically-derived) of disease-derived CMTs, reduction in contractile force was observed when compared to CMTs generated from WT hiPSCs. However a more significant reduction (more than 50%) was found in patient-derived CMTs, raising the possibility that additional genetic variants affect the function of titin and thus the emulation of pathophysiology. Similarly, there was an observed reduction in contractility of the genetically-edited CMs when compared to WT, however it was not as significant as the patient-derived CMs. The limited change in functionalities of the gene-edited CMs demonstrates the role of genetic background in the manifestation of cardiomyopathy. Similarly, Zhao et al. incorporated the Biowire II model to study left ventricular hypertrophy (LVH), through generation of cardiac tissues with LVH patient-derived hiPSC-CMs [98]. However, with the cause unknown for the underlying phenotype of LVH, chronic electrical conditioning protocols were also applied to the tissues to better mimic the clinically-observed increases in cardiac workloads in patients with hypertension. After 8 months of electrical conditioning, significant upregulation in gene expression for hypertrophy and heart failure were identified in biowires formed from LVH-patients in comparison to non-affected controls. Therefore, through use of both patient-derived cardiac tissues and external conditioning, recapitulation of pathophysiology was achieved within this 3D cardiac tissue model. Overall, the aforementioned studies demonstrate the utility of 3D diseased cardiac tissue models to recapitulate the corresponding pathophysiology, and their potential to serve as platforms for more precise cellular-level mechanistic studies.

## Conclusion and general perspective

Microengineered 3D cardiac models enable extensive mechanistic and functional studies, that prove to be limited in conventional disease testing through 2D assays or animal models. In addition, stem cell differentiation techniques provide capabilities for patient-specific disease modeling to further advance physiological relevance of engineered cardiac tissues. Gene-editing techniques, such as CRISPR/Cas9 and modRNA, could be also incorporated with these models to induce diseased CMs with specific mutations to study related pathologies, and/or to study potential disease correction through restoration. Through the technologies, modelling of genetically-derived cardiac diseases has flourished, resulting in the development of many useful platforms to better understand myocardial pathology. Despite the significant findings, there are limitations yet to be addressed, such as lack of incorporated vasculature, use of monoculture, and state of CM immaturity, that may challenge the clinical relevance of existing models. The combination of currently implemented strategies, including 2D in vitro assays and animal models, with 3D microengineered models has great potential to reveal novel discoveries that could lead to effective therapies to help prevent and/or reverse CDs.

## Abbreviations

2D: 2-dimensional
3D: 3-dimensional
BS: Brugada Syndrome
BTHS: Barth Syndrome
CD: Cardiac disease
CF: Cardiac fibroblast
CM: Cardiomyocyte
CMT: Cardiac microtissue
CPVT1: Ccatecholaminergic polymorphic ventricular tachycardia type 1
CRISPR: Clustered regularly interspaced short palindromic repeats
EAD: Early after-depolarization
ECM: Extracellular matrix
EHT: Engineered heart tissue
GAA: Acid alpha-glucosidase
GelMA: Gelatin methacrylate
HCM: Hypertrophic cardiomyopathy
hESCs: Human embryonic stem cells
hiPSCs: Human induced pluripotent stem cells
hPSCs: Human pluripotent stem cells
LCA: Left coronary artery
LQTS: Long-QT Syndrome
LQTS1: Long-QT Syndrome type 1
LVH: Left ventricular hypertrophy
MI: Myocardial infarction
modRNA: Synthetic chemically modified mRNA
mPAD: Microarray post detector
MTF: Muscular thin film
MYBPC3: Myosin-binding protein C cardiac isoform
PDMS: Polydimethylsiloxane
RyR2: Ryanodine receptor type 2
TAZ: Tafazzin
TS: Timothy Syndrome
TTNtv: Titin-truncating variant
WT: Wild type
ZDF: Zucker diabetic fatty
